# A Model of Proteostatic Energy Cost and Its Use in Analysis of Proteome Trends and Sequence Evolution

**DOI:** 10.1371/journal.pone.0090504

**Published:** 2014-02-28

**Authors:** Kasper P. Kepp, Pouria Dasmeh

**Affiliations:** Department of Chemistry, Technical University of Denmark, Kongens Lyngby, Denmark; Universidad de Granada, Spain

## Abstract

A model of proteome-associated chemical energetic costs of cells is derived from protein-turnover kinetics and protein folding. Minimization of the proteostatic maintenance cost can explain a range of trends of proteomes and combines both protein function, stability, size, proteostatic cost, temperature, resource availability, and turnover rates in one simple framework. We then explore the *ansatz* that the chemical energy remaining after proteostatic maintenance is available for reproduction (or cell division) and thus, proportional to organism fitness. Selection for lower proteostatic costs is then shown to be significant vs. typical effective population sizes of yeast. The model explains and quantifies evolutionary conservation of highly abundant proteins as arising both from functional mutations and from changes in other properties such as stability, cost, or turnover rates. We show that typical hypomorphic mutations can be selected against due to increased cost of compensatory protein expression (both in the mutated gene and in related genes, i.e. epistasis) rather than compromised function itself, although this compensation depends on the protein's importance. Such mutations exhibit larger selective disadvantage in abundant, large, synthetically costly, and/or short-lived proteins. Selection against increased turnover costs of less stable proteins rather than misfolding toxicity *per se* can explain equilibrium protein stability distributions, in agreement with recent findings in *E. coli*. The proteostatic selection pressure is stronger at low metabolic rates (i.e. scarce environments) and in hot habitats, explaining proteome adaptations towards rough environments as a question of energy. The model may also explain several trade-offs observed in protein evolution and suggests how protein properties can coevolve to maintain low proteostatic cost.

## Introduction

With vast amounts of genomics and proteomics data now available, there is an urgent need for more accurate and detailed general laws governing life, notably concerning cell cycles, reproduction and survival choices, disease states, and correlating genotype to phenotype, including the complex effects of post-translational processing, protein-protein and gene-protein interactions in living cells.

One possible unifier of life processes is *energy*: As formulated already by Schrödinger [1], life is thermodynamically distinct, with constantly renewed high-quality free energy required for building, maintaining and reproducing its complex biological structures under dispersion of heat [2], [3]. One might expect this tendency to reveal itself in the proteomics data and possibly, to provide a rationale for the many correlations that are now emerging from these data.

Another possible unifier is *evolution*, the process ultimately responsible for shaping proteomic properties. Although different proteins may be under different selection pressures relating to their specific functions and properties [4], [5], universal selection pressures indeed operate on all proteins [6]–[8], e.g. to optimize translational efficiency [9], [10], to maintain the correct functional state and stability (Δ*G*) [11]–[13], or to reduce the burden of misfolded and unfolded proteins [14]–[16]. Some degree of universal selection is evident from evolutionary rates of sequences correlating with a range of properties, notably protein expression levels that can span 5–6 orders of magnitude [17] (the expression level and evolutionary rate, or E−R, anti-correlation), observed for both prokaryotes [18] and eukaryotes [19], including mammals [20], [21]. Such evidence has led to new efforts with the goal of uncovering universal selection pressures acting on proteomes using fundamental biophysical models [11], [22]–[24], which provide a bottom-up alternative to the brute-force of the equally necessary whole-cell models [25].

One can classify the proposed universal selection pressures into three categories. First, proteins should maintain their functional state (usually a folded native state) to be functional [26] but are modestly stable (20–60 kJ/mol [27]). For a two-state unfolding mechanism [28], the contribution to the total fitness (*Φ*) of an organism arising from one particular protein *i* would thus be proportional to *P*_F,i_, the fraction of its folded, functional copies within the cell [11]: 
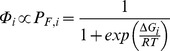
(1)where Δ*G*_i_ is the free energy of folding, *R* is the gas constant, and *T* is temperature. *Φ*_i_ should be multiplied by appropriate constants, including the total abundance *A*_i_ = *U*_i_+*F*_i_ (the unfolded and folded copy numbers of protein *i* per cell) and various cell-specific parameters. Selection for thermodynamic stability, when combined with ΔΔ*G*-distributions for arising mutations [29], explains the marginal stability of proteins without any special adaptation and accounts for fitness effects in viruses [11], [13].

Second, the E−R anti-correlation [14], [16] has been previously explained as a selection against the toxicity of misfolded proteins in the cell [16], [22]. Highly expressed proteins would then be under a stronger selection pressure since *U*_i_ scales linearly with *A*_i_ for a given stability. *Φ*_i_ can then be written in protein-specific notation [16], [22]: 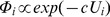
(2)


Here *c* is an unknown but empirically accessible universal fitness cost of one misfolded protein [16], [22].

Third, sequence bias towards lower biosynthetic cost of amino acids [30], [31] and lower cost of gene expression [32] are found in all domains of life [33], i.e. some selection acts to reduce the synthetic cost of a protein *I* (*E*_s,i_) [32]. Protein synthesis accounts for ∼20% of resting energy expenditure in man [34], [35], ∼30% in the larvae *Sciaenops ocellatus* [36], up to 80% in fish [37], 20–30% in grass [38], and typically ∼75% in growing microorganisms [39]. Protein degradation may cost 1/5 of the mammalian total energy expenditure [40], making protein production and clearance the most energy-consuming processes in many organisms. Thus, it seems warranted to investigate how the energy costs of proteostasis affect cell survival and reproduction, and consequently, fitness and evolution.

Despite the progress in understanding universal selection pressures, many challenges remain. First, the three types of selection suggest different molecular modes of action: one represents selection for correct protein fold, one selection against misfolded copies, and one selection against proteome synthesis costs. Second, the protein's functional profiency (e.g. *k*_cat_/*K*_M_) has not so far been coupled to these properties. Third, the concept of misfolding toxicity, probably inspired by diseases involving misfolded peptides or proteins, often lacks well-defined toxic modes of the overexpressed and misfolded proteins [41]–[43]. Fourth, since protein-synthesis costs can be of similar size as costs associated with managing misfolded proteins [44], both properties should be accounted for. Fifth, the roles of cell physiology and proteome properties and the relative strength of the selection acting on the different properties are unclear. For example, the specific fitness cost, the *c* parameter in Equation 2, must somehow be related to the physical reality of cellular processes.

In this paper, the above-mentioned concepts are combined into one function of the cellular proteostatic energy cost, derived from steady-state protein turnover kinetics and thermodynamics of protein folding. Subsequently, we show that minimization of this energy cost function can explain several proteome-wide trends. Furthermore, we explore the *ansatz* that evolutionary fitness is proportional to offspring (or cell divisions) produced per time unit, which again is proportional to the energy left for reproduction. High-quality disposable energy is central for life [39] and perhaps the main quality that defines it, and the fitness of any organism, in the strictest sense the produced offspring, should if anything scale with the energy available for this purpose.

The model unites for the first time selection acting on function, stability, biosynthetic cost, and turnover rates, includes temperature and metabolic activity, and is consistent with known trends in proteomic data relating to size, abundance, cost, evolutionary rate, and turnover. The model provides quantitative relations that can be used to evaluate the relative importance of selection for these properties and provides possible answers to observed trade-offs occurring in natural and laboratory evolution. Finally, the model allows inclusion of compensatory expression of isoforms and other genes related to the mutated protein, i.e. epistasis.

## Methods

### Protein homeostasis model

First, the total energy expenditure per time unit of an organism (d*E*_t_/dt) is considered equal to the energy produced (d*E*_p_/dt) minus the savings rate of energy, *S*: 
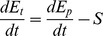
(3)


For simplicity we assume no saved energy, i.e. *S* = 0. During growth (e.g. the OX phase in yeast), if committed to reproduction, the cell will divide once enough energy is available. However, variations in *S* may result from survival strategies, cell cycle phases, etc. to be investigated in future work and omitted here for simplicity.

The proteostasis of protein *i* is now described by the simple kinetic model: 
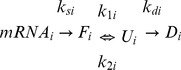
(4)


Here, *mRNA*_i_, *F*_i_, *U*_i_, and *D*_i_ signify mRNA, folded, unfolded or misfolded, and degraded copies of protein *i* in the cell. Correspondingly, *k*_si_, *k*_1i_, *k*_2i_, and *k*_di_ are the rate constants of synthesis, unfolding, folding, and degradation of this protein. The model resembles previous models [10], [45], but expands degradation to act on misfolded copies and transcriptional and translational processes are considered constant, since we are concerned here with the selection acting on the protein product. While nucleotide substitutions may also affect translation speed and accuracy [46], which is compatible with selection for energy-cost minimization [16], the focus on the protein product is justified by recent work showing that protein concentrations are more strongly regulated and most likely under stronger selection pressure than corresponding mRNA levels [47]. The model is also in line with the recent findings by Shakhnovich and co-workers that fitness depends on protein turnover acting on intermediates in an “active cytoplasm” where the protein turnover variables may change [48]. The rates of change in *F*_i_, *U*_i_ and *D*_i_ at steady state are: 
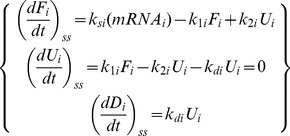
(5)


Here, the change in *U*_i_, with its abundance being typically 10^−6^ *A*_i_ or less, is ∼0, giving *k*_1i_ *F*_i_ = (*k*_2i_+*k*_di_) *U*_i_. Mass conservation 
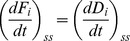
 yields after rearrangement *k*_si_ = 2 *k*_di_ *U*_i_/(*mRNA*_i_). A typical value of *mRNA*_i_ is 10^−4^ *A*_i_ [49]. The ratio of folded to unfolded copies is: 
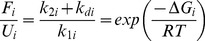
(6)


Δ*G*_i_ is the free energy of folding of protein *i*. *k*_si_ reflects the slowest process of protein synthesis (often the folding process) [50]. However, for small proteins, typical refolding *k*_2i_ are 10^1^–10^5^ s^−1^ [51], i.e. ribosomal chain elongation (∼15 aa/s, typically 10^−2^ s^−1^) becomes rate-limiting. The average half-life of proteins in yeast implies an average *k*_di_ of ∼0.016 s^−1^ [52]. The probability of a protein being folded is: 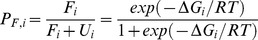
(7)


### Proteostatic cost function and fitness as chemical energy available for reproduction

We now invoke the *ansatz* that the fitness *Φ* of an organism is proportional to the offspring (or cell divisions) produced by the organism per time unit, which again is proportional to the chemical energy left for reproduction per time unit, d*E*_r_/dt. This term can be expressed by the total energy produced (and thus, consumed) minus the energy used to maintain basal processes, d*E*_m_/dt: 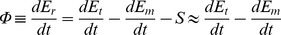
(8)


Here, d*E*_t_/dt is the total metabolic rate of the organism. In the comparison of two individuals, all-else-being-equal, the one with the proteome that requires the smaller maintenance energy will have more energy available for reproduction and will thus have higher relative fitness. Fitness approaches zero as d*E*_t_/dt≈d*E*_m_/dt, interpreted as the point of entering a dormant phase (e.g. the *G*_0_ phase for yeast or sporulation for diploid cells) and shifting to full maintenance [53]. As proteostasis consumes most of the chemical energy available to the organism [34], [37], [40], we consider other costs *C* such as RNA metabolism and ion pumps constant. d*E*_m_/dt is divided into the energy used for protein synthesis *E*_s_ and degradation *E*_d_ of the proteome per unit time, with regulation costs such as post-translational modification contained within these: 
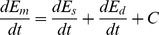
(9)


Using the kinetic scheme (4) and (5), we now write: 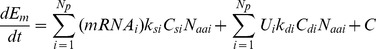
(10)


*N*_aai_, *C*_si_, and *C*_di_ are the number of amino acids in protein *i* and the synthetic and degradation cost (in units of phosphate bonds) of an average amino acid in protein *i*, and *U_i_* = 
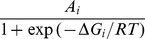
. Using *k*_si_ = 2 *k*_di_ *U*_i_/(*mRNA*_i_), the total proteome fitness is the summed contribution of all *N*_p_ proteins: 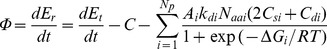
(11)


Equation 11 was derived from our *ansatz* assuming steady state, that non-proteome costs are separable from proteome costs via *C*, and that mainly non-native states are degraded. The fitness function scales with the energy left for reproduction, expressed as the remaining energy after proteome expenditure per time unit.

### The selection coefficient

Arising mutations can potentially change one or more protein properties. An arising mutant with fitness *Φ*′ has a selective advantage/disadvantage, *s*′ = (*Φ*′−*Φ*)/*Φ*, where *Φ* is the fitness of the prevailing variant (wild-type), giving: 
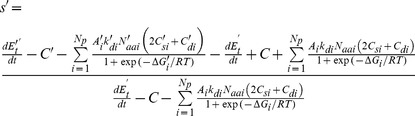
(12)


Importantly, for a single, arising mutation in one protein *i*, all other phenotypes and properties, the total metabolic rate, and non-proteome costs *C* cancel out: 
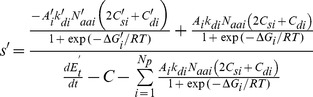
(13)


As described below, epistasis can be described explicitly by modifying the parameters of additional proteins connected to the mutated protein *i* in the general Equation 12, but to illustrate the mechanics of the model, we consider Equation 13 in the following. A mutation in a protein *i* could in principle affect any of the properties in Equation 13: If *N*′_aai_≠*N*_aai_, the mutation would be an indel. The amino acid cost (which does not need to be simply the precursor cost but can be the full synthetic cost per copy of the specific protein) would be adjusted by *C*′_si_−*C*_si_. If the mutant is harder to degrade, *k*_di_ would decrease, etc.

## Results

### Selection against misfolded or unstable protein copies

As a first result, we show that previously proposed mechanisms of selection acting to preserve protein stability [11], [13] or prevent misfolding [14], [16] are special cases of Equation 13 and we resolve the previously proposed empirical fitness cost parameter [16], [22] into its fundamental proteostatic variables. In the following, the amount of *U*_i_ should strictly imply “nonfunctional” (not misfolded), as e.g. intrinsically disordered proteins are functional without a well-defined native state. To see the correspondence to previous findings, in the special case that only Δ*G*_i_ changes for one protein *i*, 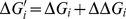
(14)the selection coefficient becomes: 
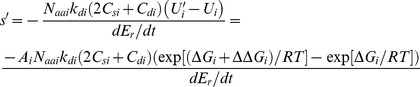
(15)


The denominator is, as seen from Equation 8 and 12, the chemical energy spent on reproduction in the wild-type. We have simplified *U_i_* slightly, as most proteins are >10-fold more stable than −RT, i.e. exp(Δ*G*_i_/RT)∼10^−4^ or less: 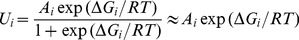
(16)


Using this expression in Equation 15, selection pressure to reduce proteome energy cost can be understood to work directly on *U*_i_, and the selective advantage is proportional to the difference between the number of unfolded (or strictly: unfunctional) protein copies that are targeted for degradation in the two variants, viz. Equation 4. Thus, previously described selection for stability [11] is a special case of Equation 15 (which is a special case of Equation 12) where all variables except stability of one mutated protein are assumed constant.

Second, or model of proteostatic cost can also be compared with previously proposed selection against *U*_i_ (unfunctional, misfolded copies targeted for degradation), called *m* in previous work [16]. In that work [16], it was assumed that any increase in *U*_i_ gives the same change in *Φ* regardless of protein *i* in question (i.e. *c_i_* was assumed universal and independent of *i*), giving the selection coefficient for protein *i*: 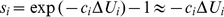
(17)


The last step follows since any realistic selection coefficient will be several orders of magnitude smaller than one. For *s*_i_<0.01, this expansion of the previously proposed Equation 2 is correct to within four digits. The corresponding expression from our model assuming that stability is the only changing property, i.e. Equation 15, is: 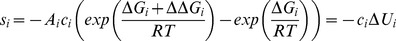
(18)


Therefore, our model recovers the previously suggested selection pressure against unfolded protein copies [16] and similar expressions expressed by folding stabilities [22]. More importantly, comparison of our Equations 13 and 18 reveals an explicit interpretation of the empirical, dimension-less cost parameter *c*_i_ [16]: 
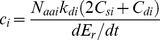
(19)


Here, *N*_aai_ is the number of amino acids in the protein, *k*_di_ is the degradation rate constant in s^−1^, *C*_si_ and *C*_di_ are the per-amino-acid costs of synthesizing and degrading the protein, and d*E*_r_/dt is the total metabolic energy devoted to reproducing the organism, as described in the Methods section.

To estimate a typical size of *c*_i_, we used a metabolic rate of ∼0.9 J s^−1^ g^−1^ for a yeast cell mass of ∼3.4×10^−11^ g at 37°C [54] and 2/3 or ∼0.6 J s^−1^ g^−1^ as the proteome respiration rate (d*E*_t_/dt−*C*) [39]. With 10% reproductive energy, this gives d*E*_t_/dt−*C* = 2.0×10^−11^ J s^−1^ and d*E*_r_/dt = 2.0×10^−12^ J s^−1^. An average yeast protein has *N*_aai_∼467 [55], and degradation costs ∼1 ATP molecule per amino acid [56], with ∼30 kJ mol^−1^ of a phosphate bond, i.e. *C*_di_∼30 kJ/mol. Synthesizing amino acids from precursors in a minimal medium costs 10–80 phosphate bonds [32], with a yeast-composition [57], weighted average of ∼26 phosphate bonds, giving *C*_si_ of ∼800 kJ/mol (less in a rich medium). Protein-chain synthesis is estimated at 11–19 ATP per amino acid [58], i.e. *C*_si_ = 330–630 kJ/mol, plus costs of ribosome maintenance and chaperones. We used a conservative *C*_si_ = 1500 kJ/mol. A typical degradation constant *k*_di_ is 2.7×10^−4^ s^−1^ (∼43 min half-life for an average protein in yeast [52]). Using these experimentally known parameters, we can then calculate the energetic selection pressure acting on a typical yeast protein. Converting from kJ/mol to J and dividing by Avogrados' number yields 
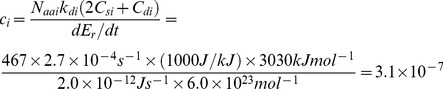
(20)


This value is for a typical yeast protein if 10% proteome energy is devoted to reproduction. Typical values of the involved parameters are given in Table 1. Due to the variations in these properties, notably *k*_di_, the value of *c*_i_ can vary by more than three orders of magnitude for different proteins *i*, i.e. the assumption [16] that this parameter is independent on the protein in question (i.e. that *c*_i_ = *c* for all proteins *i*) is not valid. These large variations in individual protein properties make sensitivity analysis less meaningful until specific parameters for individual proteins can be used directly in the model to test the model's implications. The reason why the fitness cost is not universal but protein-dependent is, simply speaking, that the selection acting against misfolded copies at any time in a cell is highly dependent on the kinetic turnover and cost of the protein *i*, since these are proportional to the proteostatic handling costs.

**Table 1:** Parameters required for calculating the fitness function, and their default values.

PARAMETER	DEFAULT VALUE	UNITS
E(ATP)	30	kJ/mol
cell mass (yeast)	3.40E-11	g
*m*_i_ (Mass of average amino acid (aa))	130	g/mol
d*E*/dt (total specific respiration rate)	0.90	J s−1 g−1
*C* (cost of non-proteome respiration)	0.30	J s−1 g−1
d*E*/dt Yeast proteome part ( = d*E*/dt−*C*)	0.60	J s−1 g−1
d*E*/dt total Proteome expenditure	2.04E-11	J s−1
*F* (Fraction of d*E*/dt spend on reproduction)	0.10	
d*E*r/dt (reproductive energy, = *F* d*E*/dt)	2.04E-12	J s−1
d*E*_m_/dt (maintanance energy, = (1−*F*) d*E*/dt)	1.84E-11	J s−1
*N*_aai_ (length of protein *i*)	467	
*M*_i_ Mass of protein with *N*_aai_ ( = *m*_i_ *N*_aai_)	60710	g/mol
*A*_i_ (copy number of protein *i* per cell)	10000	
Δ*G*_i_ (free energy of folding, protein *i*)	37	kJ/mol
*mRNA*_i_ = *A*_i_/4800 (mRNA level)	20.8	
*R* (rate of chain synthesis aa/s)	15	s−1
*k*_ribosome_ ( = *R*/*N*_aai_)	3.21E-02	s−1
*k*_di_	2.69E-04	s−1
*C*_si_ (synthetic cost per aa in protein *i*)	1500	kJ/mol
*C*_di_ (cost of degrading avr. aa)	30	kJ/mol
*k*_fi_ (folding rate constant)	1.00E-05	s−1
*k*_si_ ( = Min of *k*_ribosome_ and *k*_fi_)	1.00E-05	s−1

### Proteostatic selection against mutations that impair protein function

In the following, we will show that selection against mutations that impair the functional proficiency of a protein, i.e. hypomorphic mutations [59], can be understood from our proteostasis model. If a protein is mutated to the effect of reduced proficiency (e.g. if *k*_cat_/*K*_M_ of an enzyme is reduced), then all-else-being equal, to maintain homeostasis, the protein would be required in more copies, i.e. *A*_i_ would increase to preserve total turnover of the affected reaction.

The reduced proficiency may be compensated by changing the expression of multiple other proteins involved in the same aspect of homeostasis as the affected protein (epistasis). In the simplest case, this occurs by increased expression of isoforms [60], or of other proteins with similar functions [61]. Also, the total expression and turnover relating to the mutated protein itself can change in the “active cytoplasm” as demonstrated recently [48]. The extent of compensatory expression of the mutated gene and of other genes (epistasis) are important to understand the full proteostatic effects of mutations in a given protein, and such compensation will be protein-specific: For highly systemic proteins, compensation may be large, as seen e.g. in sickle cell disease where hemoglobin mutations reduce the oxygen-carrying ability of the protein and substantially increase the protein's expression [62], [63], or in cancers where mutant p53 are subject to higher expression levels [64]. This leads to increased proteostatic costs, because of the larger *A*_i_ required to maintain critical functions. If a mutation almost completely impairs an essential protein (i.e. an amorphic mutation [59] of a systemic protein), the individual will be purged from the population either because compensatory expression may be so energy-consuming that the organism cannot maintain itself, or because of the absence of the protein function itself. In contrast, mutations in less important proteins will involve limited compensation, with dormant genes as the extreme examples.

Importantly, these effects can be directly included in our general fitness function (Equation 11) and the associated selection coefficient (Equation 12), by changing the abundance of the additional, affected genes. However, since these effects are protein-dependent but directly includable in the model, we will not consider such variations in the following. To show that function-impairing mutations can be selected against due to energy costs, we thus ignore epistasis, assuming that all other proteins are unaffected, i.e. reducing Equation 12 to Equation 13. However, it is clear from Equation 11 and 12 that compensatory epistasis of hypomorphic mutations will also increase proteostatic costs via larger abundances *A*_j_ of protein(s) *j* connected to the mutated protein *i*.

We will show below that selection of function-affecting mutations can be affected by proteostatic energy costs associated with the mutation rather than the impaired function itself, and that such selection can explain the conservation of abundant proteins. The increased expression of a hypomorphic mutant will incur a fitness cost not only due to function itself but also due to less available chemical energy, providing a general contribution to the selection against function-impairing mutations that should probably be considered in protein evolution.

### Highly expressed proteins are under stronger proteostatic selection

**Figure 1:**
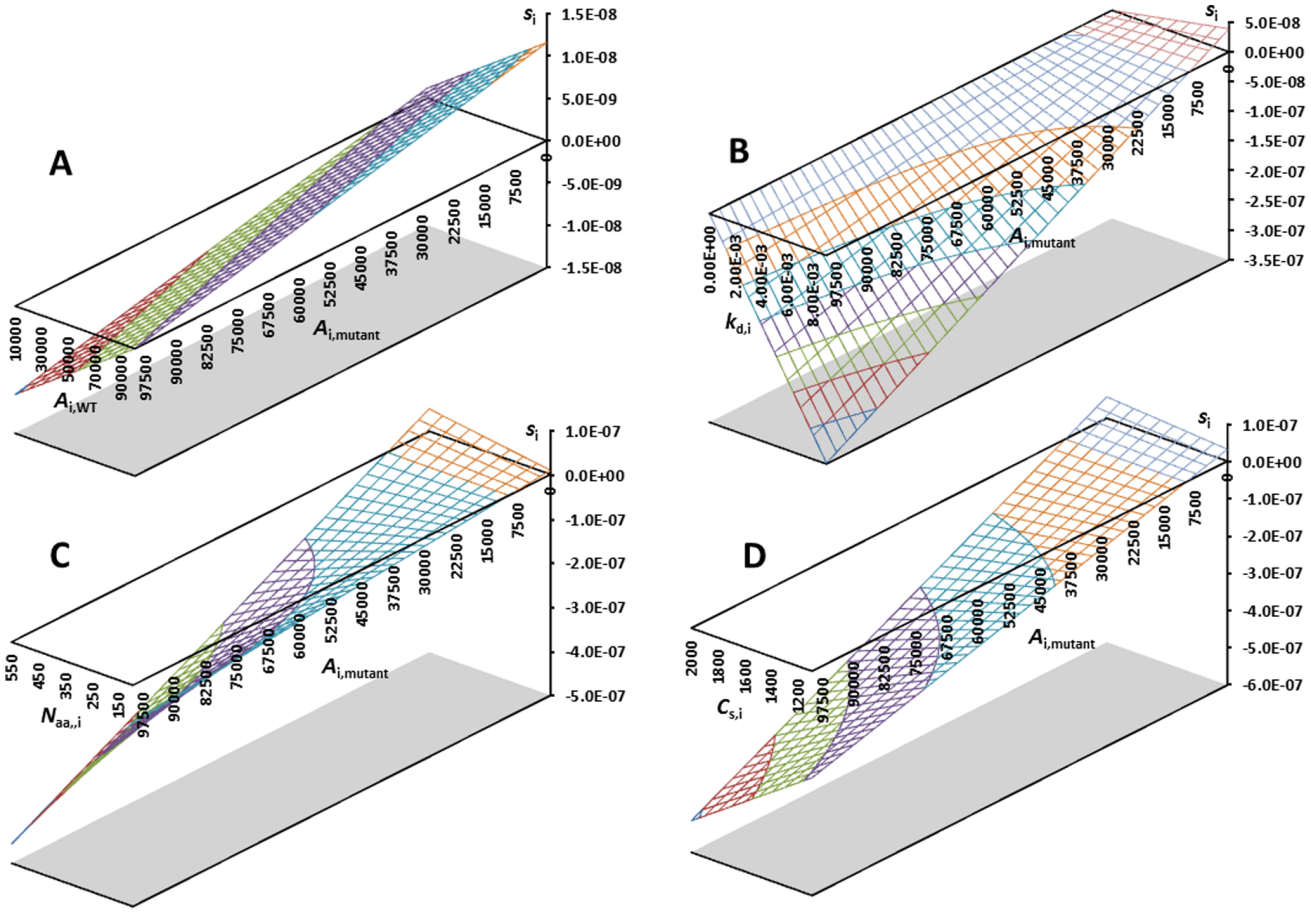
Selection spaces *s*_i_ (fitness-differences normalized to wild-type fitness) for mutations causing increased mutant protein expression (*A*_i,mutant_). (**A**) Selection acts against increased protein abundance of mutant vs. wild-type (*A*_i,WT_) (Default values of parameters from Table 1). (**B**) High-turnover proteins (with large values of *k*_d,i_) are under stronger selection pressure to perform optimally. (**C**) For a high-turnover protein (life time ∼1 minute, *k*_d_ = 0.01 s^−1^, larger proteins are under stronger selection to perform optimally, *ceteris paribus*. (**D**) Selection pressure is stronger for proteins that are synthetically expensive, as measured by *C*_s,i_ (*k*_d_ = 0.01 s^−1^).

Figure 1 shows a “selection landscape” (relative fitness landscape normalized to wild-type fitness) of *s*_i_, computed from our model (Equation 13) as a function of changing properties of protein *i*, with all other properties of the proteome being constant. Normalization by the wild-type fitness was done using the 2×10^−12^ W used for reproduction in our model yeast cell. When selection coefficients are close to zero, the effect of a mutation is nearly neutral. The protein in this case has average size, stability, and turnover properties. Figure 1 shows the impact of mutations where the wild-type abundance *A*_i_,_WT_ is changed, e.g. in response to functionally impairing or improving mutations. The space covers the range of abundances typically encountered in a yeast cell (0–100,000). Figure 1A displays the general proteostatic selection acting on mutations that cause changed expression, for a variable WT abundance, *A*_i_,_WT_, using the default values of Table 1. Figures 1B, 1C, and 1D all display results for one typical WT abundance, *A*_i_,_WT_ = 10,000.

Figure 1A shows a simple linear increase in selection pressure as WT and mutant abundances differ. For a typical, well-expressed yeast protein of *A*_i_ = 10,000, a mutation that reduces *k*_cat_ 10-fold giving 10-fold higher abundance, *ceteris paribus*, would carry a proteostatic selective disadvantage of −10^−8^. However, as Figures 1B–1D show, such a protein will be under stronger selection if the term *A*_i_×*N*_aai_×*k*_di_×(2*C*_si_+*C*_di_) is larger than average. A highly expressed protein (copy number 100,000) that has its functional proficiency impaired by only 10-fold would require 900,000 additional copies of itself to maintain homeostasis, causing highly expressed proteins to be more conserved, because many more of their arising mutations would reduce the chemical energy available for reproduction. Using the same parameters as in Figure 1 and Table 1, such a protein would have a selective disadvantage of 10^−7^, similar to typical effective population sizes even with other protein properties being average. Since stronger selection against deleterious mutations leads to increased conservation of amino acids, an E-R anti-correlation arises naturally from our model.

In reality, all the properties of a protein will change upon mutation: stability and proficiency will change, as will expression, turnover, and proteostatic precursor cost per protein. As described recently, stability and abundance both affect evolutionary rates and act together via mutation-selection balance to keep selection pressures more independent of expression levels [22]. This important mechanism was seen in evolutionary simulations but is consistent with our deduced selection pressure that grows with abundance but decreases with stability of the protein, viz. Equation 15. The empirically confirmed [22] anti-correlation between Δ*G* and ΔΔ*G* of fixated mutations follows already from the fact that more stable proteins are, for the same expression level and other parameters being similar, under less selection pressure (Equation 15) i.e. they can accept more deleterious mutations with larger ΔΔ*G* values. In Protherm, ΔΔ*G* values of mutations are however not the result of natural evolution but protein engineering. The reason for the anti-correlation in Protherm [22] may be due to the fact that less stable proteins can accept less destabilizing mutations also in the laboratory where all proteins are under stability constraints relating to the expression protocol. Since our fitness function reduces to that of Ref. 22 in the limit where only stability changes upon mutation, our model is consistent with these findings, although the cause of selection (the phenotype actually selected for) is energy, not stability.

Thus, our model can explain one of the most persistent correlations in proteomics, that between evolutionary conservation and expression level, and it unifies in one framework mutations that affect protein function, stability, turnover, and handling costs allowing estimates of their relative importance, while also accounting for epistasis (the full Equation 12). For systemic proteins with full (same-gene or via epistasis) compensation, proteostatic selection can be substantial: If a mutation reduces *k*_cat_ or increases *K*_M_ of an enzyme by 10-fold, which is quite feasible as it involves only a few kJ/mol of changing activation or substrate binding free energies, to preserve steady-state turnover, the copy number of the mutant and its associated genes would need to be ten times higher in the simplest case, i.e. even apparently subtle functional mutations can involve proteostatic fitness costs large enough to affect selection. Such a high cost can hardly be realized by the cell, and thus, compensatory expression will be incomplete, so that the cell suffers a combination of increased proteostatic costs and decreased overall protein function. Homeostasis may then be adjusted to the lower proficiency of the mutated protein as far as the mutated protein is connected to other protein functions.

### Proteostatic selection on short-lived proteins

From Figure 1B, disadvantageous mutants of proteins with shorter life times (larger *k*_d_) will be more strongly selected against. Thus, many regulatory proteins that are highly connected in a network sense will tend to be more conserved, not necessarily because they are more connected but also because they have high turnover rates (viz. Equation 13). For example, *E. coli* transcription factors that are highly connected in networks have fast turnover despite being highly expressed [65], and such proteins will be under substantial selection pressure according to our model, which thus provides a new mechanism behind the evolutionary conservation of some highly connected proteins. In fact, the nature of the actual fitness reduction causing conservation of connected proteins is not very tangible but becomes very tangible when considering the energetic consequences of short-lived proteins suddenly required at multiple-fold higher mutant levels.

### Selection on larger and more synthetically costly proteins

Figure 1C and 1D show the selection coefficients of the same typical protein with mutations ranging from beneficial (hypermorphic), giving lower expression than 10,000, to impairing (hypomorphic), giving higher expressions, up to 100,000, now with variable protein length (*N*_aai_) and protein synthesis cost per amino acid (*C*_si_). Again, compensatory expression is used here to illustrate the nature of the selection pressure, and the actual magnitude of the partial compensation and epistasis effects can be accounted for in specific proteins via Equation 12 by adjusted the abundances *A*_i_, *A*_j_, etc. of involved proteins after mutation.

In accordance with Equation 13, selection pressure increases with protein size and synthetic cost, so that a smaller fraction of typical arising mutations are nearly neutral for the larger and more expensive proteins. The model explains the experimental observation that for typical yeast proteins with *N*_aai_>250, larger proteins are more conserved [66] (although for the minority of proteins smaller than *N*_aai_∼250, the reverse is seen). The model captures this effect for the majority of proteins, since slower evolution in most (normal-sized and large) proteins results from stronger selection against typical hypomorphic mutations, because larger proteins are more proteostatically expensive, all else (notably expression levels) being equal, i.e. compensatory expression is more costly. For small proteins, the reverse positive correlation probably arises from the smaller size-range and the relatively fewer sites that do not affect function directly, although this requires more investigation. As seen from Figure 1D, the model also explains why there is a bias in protein sequences across all three domains of life towards synthetically cheaper amino acids [30]–[33]: Selection for proteome energy makes any typical hypomorphic arising mutation more strongly selected against when *C*_si_ is higher, since compensatory expression of the mutant will be more costly due to the more expensive amino acids involved in the protein in general. Since the typical arising mutation is hypomorphic, a larger fraction of such typical mutations will be purged in the proteostatically costly (large, abundant, expensive, short-lived) proteins, causing an anti-correlation between evolutionary rate and these properties.

### Fixation probabilities of arising mutations

In the following, we take this discussion a step further by computing the probability of fixating mutations depending on their biochemical properties. The rate of evolution scales with the mutation rate times the fixation probability *P_fix,i_* of new arising mutants, which again increases with their selective advantage [67]: 
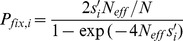
(21)where *N*_eff_ and *N* are the effective and census population sizes. Positive selection, if strong enough to lead to fixated mutants, will increase stability to reduce *U*_i_ costs, consistent with previous findings [11].

**Figure 2:**
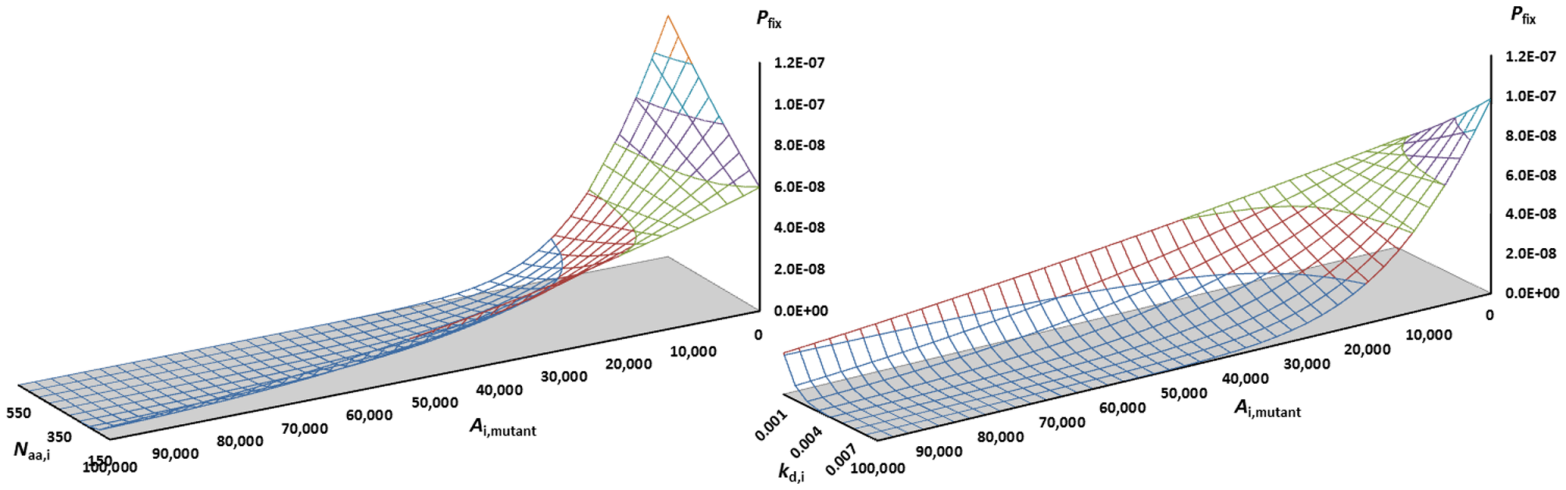
Fixation probabilities as a function of protein properties for a typical yeast population (*N*_eff_ = 10^7^). A mutation of a wild-type protein with *A*_i_ = 10,000 leads to compensatory altered mutant expression *A*_i,mutant_ to maintain homeostasis. Fixation probabilities are shown for variable protein size (left) and degradation rate constant (right).

Figure 2 shows the nonlinear region of the fixation probability space for mutations that lead to changed expression vs. variable protein length and turnover constants, calculated with *N*_eff_ = 10^7^, corresponding to the selection spaces in Figure 1B and 1C. The probability of fixation increases as less mutant protein is required in beneficial mutations (WT abundance  = 10,000 copies), and the probability increases faster for larger and short-lived proteins. These proteins are in turn less likely to accept impairing mutations that lead to increased protein expression, due to the cost selection against them. Since evolutionary rates are proportional to fixation probabilities, this implies that larger or short-lived proteins are more evolutionary conserved near fitness optimum where impairing mutations dominate, but will evolve faster if (less likely) beneficial new mutations occur.

### Stability effects of typical mutations directly affect fitness via proteostatic energy costs

Until now, we have discussed general mutations that change the functional proficiency of the protein, leading to compensatory increased or reduced protein expression. In the following, we discuss how stability-changing mutations can affect proteostasis. This is quite relevant since mutations on average are significantly destabilizing (typically by ∼5 kJ/mol [13]).

The average Δ*G*_i_ of a yeast protein can be assumed to be −37 kJ/mol at 37°C [68]. An abundant protein (*A*_i_∼100,000) of average stability has 0.037 unfolded copies at steady state. A typical arising mutation would destabilize by ∼5 kJ/mol and ΔΔ*G*_i_>12 kJ/mol may occur in ∼15% of arising mutations [29]. For such a mutation, there would be ∼4.5 unfolded copies at any time during steady state (in comparison, the total 50 million proteins of average stability per cell give ∼19 unfolded copies at steady state). When this Δ*U*_i_ = 4.5 is multiplied by *c*_i_, the selection disadvantage passes 10^−6^, or 10-fold the inverse, typical effective population size *N*_eff_ of yeast (∼10^7^) [69], and similar to the empirical estimate for an unfolded protein copy (∼10^−6^) derived from growth-retarded yeast mutants carrying nonfunctional, misfolding protein [70]. Our model thus recapitulates experimentally observed selection against misfolding and explains it as due to proteome cost minimization, with no explicit misfolding toxicity.

**Figure 3:**
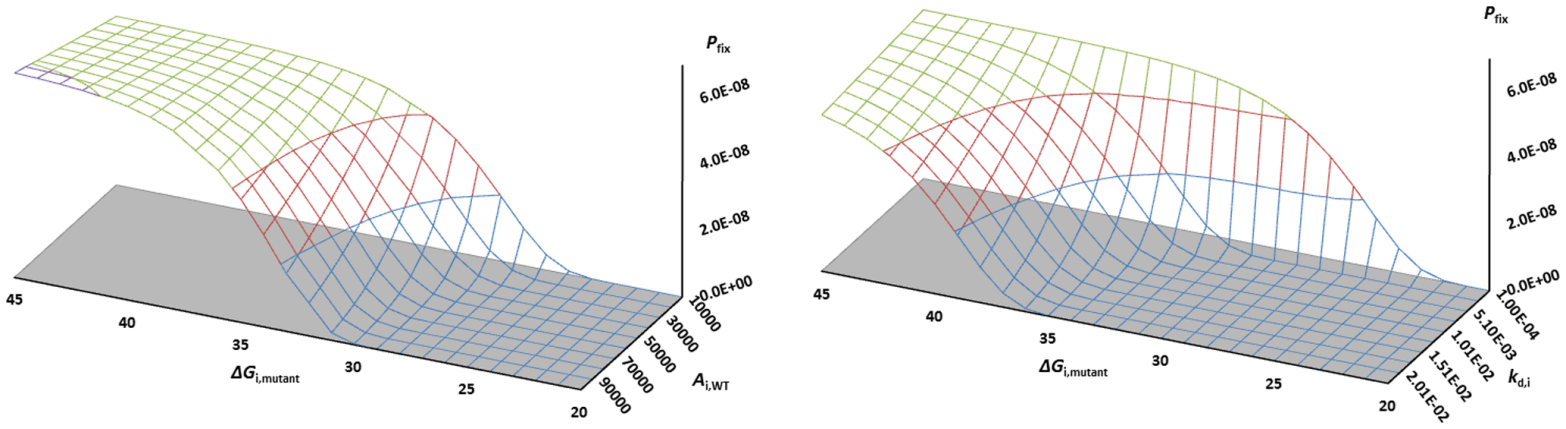
Probability of fixation of arising mutations vs. their stability. The plots are calculated for an average yeast protein with *N*_eff_ = 10^7^ and a stability of 37 kJ/mol Left: Fixation of mutants vs. the abundance of the protein. For most common, destabilizing mutations, abundant proteins evolve slower by several factors (viz. mutants at ∼30 kJ/mol). Right: Fixation vs. the turnover constant of the protein. Proteins with short life times (large *k*_d_) have nearly zero fixation probability for most common mutations, whereas long-lived proteins accept mutations more often.

The fixation probabilities of mutations that affect stability are shown in Figure 3 using the parameters of Table 1. The chart to the left shows the dependence as a function of protein abundance, and the chart to the right shows the dependence on turnover (*k*_d_) for *A*_i_ = 10,000. The typical ∼5 kJ/mol-destabilizing mutations (shown at 32 kJ/mol stability of the mutant) are selected against in more abundant proteins, causing their fixation probabilities to approach zero, while in less abundant proteins, such mutations are accepted with rates resembling neutral evolution (∼1/*N*_eff_). Thus, not only functional mutations, but typical mutations regardless of functional effect, since these are on average destabilizing, will cause more abundant proteins to evolve more slowly, confirming previous explicit simulations [11], [22] but explaining these in terms of fundamental proteostatic parameters. Thus, we find that highly abundant proteins are more evolutionary conserved for two reasons: Typical arising mutations are destabilizing enough to be more selectively disadvantageous in more abundant proteins, due to the increased cost of managing the less stable mutant, and function-impairing mutants will be more selected against in abundant proteins where the compensatory expression cost is larger, causing typical arising mutations to be more often purged (thus slowing evolutionary rates) in abundant, costly, large, and short-lived proteins.

### Evolution of protein stability: Correspondence to experimental distributions

We then investigated whether our model's selection against proteostatic costs can reproduce the well-known empirical distribution of protein stabilities, which are skewed Gaussians or bi-Gaussians with average stabilities of the order of −5 to −8 kcal/mol and with the distribution tailing towards higher stability [11], [13], [29], [68]. To this aim, we used an iterative numerical algorithm to compute the final distribution of stability of proteins when their fitness is quantified by Equation 11.

The distribution of protein stabilities is a limiting distribution under mutation-selection balance, i.e. typical, destabilizing mutations occurring by random drift are countered by more stabilizing mutations with increased fixation probability after the stability has been reduced by such drift [7], [8], [11], [12]. The specific characteristics of the distribution (i.e., shape and different moments) thus depend on parameters such as the distribution of fitness effects and *P*_fix_ of arising mutations. The ΔΔ*G* value of each arising mutation was sampled from the distribution of mutational effects on protein stability (ΔΔ*G* distribution) with the following bi-Gaussian function [29]: 
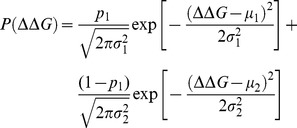
(22)where *p*_1_ is a weight factor of the first Gaussian and (1−*p*_1_) is a weight factor of the second Gaussian, roughly corresponding to core and surface amino acids of the protein, μ_1_ and μ_2_, are the average values of each Gaussian function, and σ_1_ and σ_2_ the standard deviations. For a typical protein, values of μ_1_, μ_2_, σ_1_, and σ_2_ of 0.56±0.12, 1.96±0.53, 0.90±0.16, and 1.93±0.29, respectively, can be used [29]. Upon the first mutation, Δ*G*_0_ is changed to a distribution of Δ*G*s with probabilities drawn from Equation 22, given the initial distribution, *P*_1_(Δ*G*). In the second mutation phase, each protein with its corresponding Δ*G* drifts toward lower stabilities caused from pure sampling (Equation 22), however, scaled by probability of fixation (Equation 21). In other words, a protein can become less stable by an arising mutation but this mutation can either be fixed in or purged from the population depending on its probability of fixation. We described transition of a protein with free energy Δ*G*_i_ in each phase to Δ*G*_j_ in the next phase by the following probability: 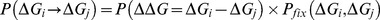
(23)


Where *P*(ΔΔ*G* = Δ*G*_i_−Δ*G*_j_) is the corresponding probability of an arising mutation with ΔΔ*G* value and *P*_fix_(Δ*G*_i_,Δ*G*_j_) is the probability of fixation of an arising mutation that changes the background stability Δ*G_i_* to Δ*G_j_*. With the initial distribution of protein stabilities, *P*_1_(Δ*G*), we can calculate the distribution of Δ*G* of the second phase from the following integral: 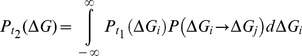
(24)


This iterative procedure was continued with t_3_, t_4_, …,t_n_ each representing one new fixed non-synonymous mutation in the population, converging to a limiting distribution of Δ*G*s.

**Figure 4:**
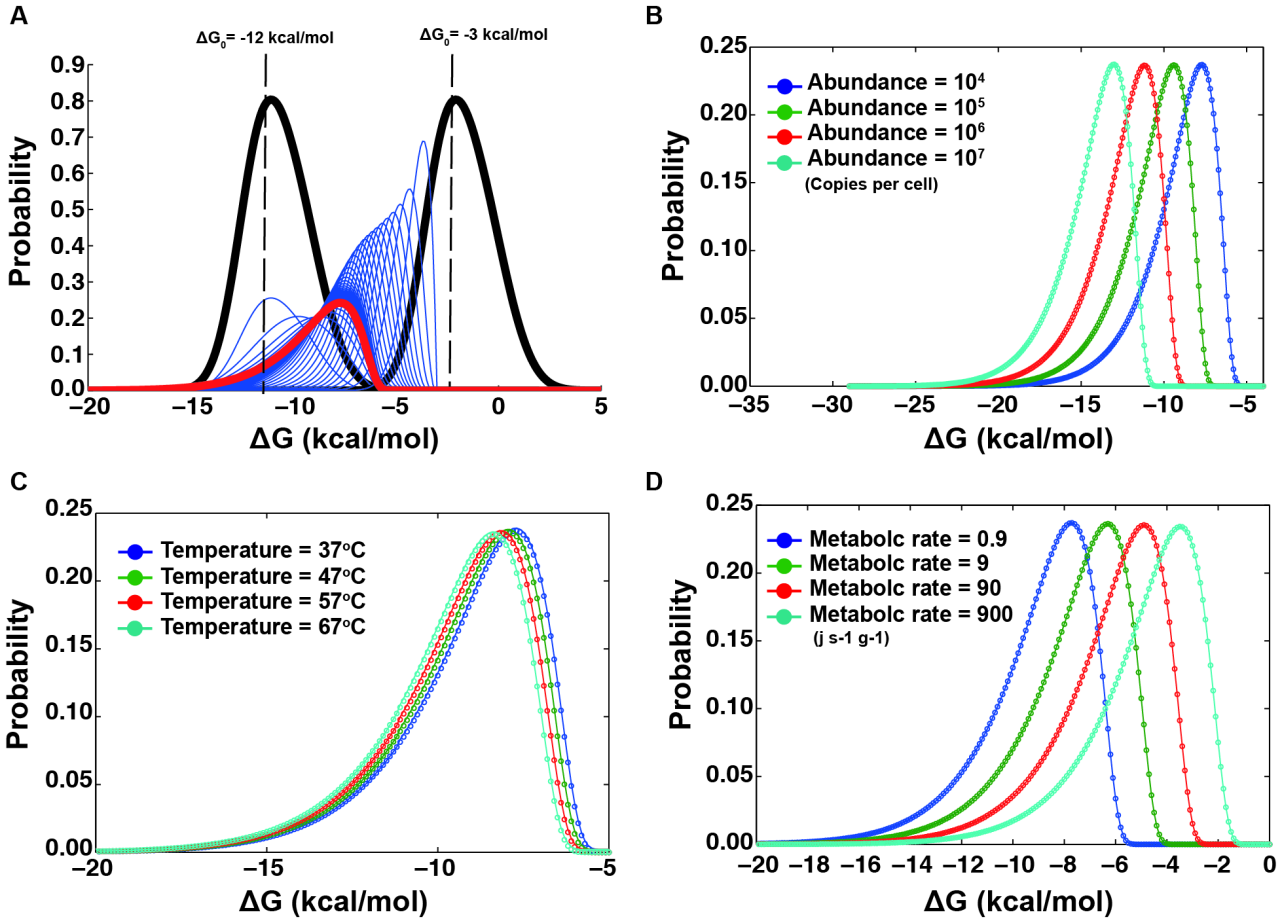
Evolution of protein stability according to the model. (**A**) Equilibrium distribution of Δ*G* obtained from an initial Δ*G* of −3 kcal/mol (red curve) and Δ*G* = −12 kcal/mol (black) via consecutive mutations (blue curves), using the fixation probability of Equation 21, the selection coefficient of Equation 15, the standard parameters of Table 1, and the iterative scheme, Equations 22–24. (**B**) Equilibrium distribution of Δ*G* for proteins with copy numbers of 10^4^ (blue), 10^5^ (green), 10^6^ (red), and 10^7^ (cyan) with a total cellular metabolic rate of 2.8×10^−2^ Js^−1^g^−1^. (**C**) Equilibrium distribution of Δ*G* for proteins at temperature of 37°C (blue), 47°C (green), 57°C (red), and 67°C (cyan), using 2^12^ copies per cell and a metabolic rate of 2.8×10^−2^ Js^−1^g^−1^. (**D**) Equilibrium distribution of Δ*G* for total metabolic rates of 0.9 Js^−1^g^−1^ (blue), 9 Js^−1^g^−1^ (green), 90 Js^−1^g^−1^ (red), and 900 Js^−1^g^−1^ (cyan) for a protein with 2^12^ copies in the cell.

Figure 4A shows the evolution of the Δ*G* distribution (in black) starting from either Δ*G*_0_ = −3 kcal/mol or Δ*G*_0_ = −9 kcal/mol for a protein with an abundance of 2^12^ molecules per cell. Both trajectories converge to the equilibrium distribution (shown in red) that peaks at Δ*G* = −6.5 kcal/mol, i.e. the sampled distribution. For both initial values, the Δ*G* distributions converge to the final distribution after ∼14 mutations, as judged from a Kolmogorov-Smirinov two-sample test. The overall shape and skewedness of the final distribution is consistent with the distribution of protein stabilities reported previously [11] and with that found empirically from the Protherm data base, but notably, it was produced here under an influence of a fitness function (viz. *P*_fix_) that has proteostatic energy cost as its main phenotype and stability as the variable protein property.

After showing the correspondence to purely stability-based fitness functions, with our model, we can now investigate how properties such as copy number, habitat temperature, and total cell metabolic rate affect the equilibrium stability distribution, as shown in Figure 4B–4D. From Figure 4B and Figure 4C, the model predicts highly expressed proteins and proteins in hot habitats to evolve to higher stabilities. Both of these results are consistent with general findings [68], [71]–[73] but importantly, in our model, selection acts on the phenotype of total proteostatic energy cost. Since we use realistic parameters for this calculation, it suggests that selection acting on thermophiles is largely interpretable as selection against increased turnover costs of denaturated proteins at higher temperatures, not against misfolded proteins *per se* [68], although the result is similar. Our model also predicts that adaptation to thermostability is dependent on the protein's proetostatic properties, e.g. abundance, size, and synthetic cost. Our model suggests that selection against misfolding is not necessarily associated with a specific toxic phenotype or loss of function of the natively folded protein, but rather with selection against the increased chemical energy costs of protein turnover following from an increase of the degradation-prone protein pool (*U*).

Figure 4D shows how the equilibrium stability distribution depends on the total metabolic rate of the cell. Cells with lower metabolic rates are predicted to exhibit a shift towards more stable proteins if the proteome is similar, i.e. with the same parameters, copy numbers, etc. From Equation 13, the selection coefficient of a newly arising mutation under such conditions is inversely proportional to the total metabolic rate of the organism. Since the total metabolic rate is restricted by energy availability in the habitat [74], selection pressure against proteostatic cost grows as resources become scarce. This finding is also fully consistent with experimental results, e.g. from adaptations towards low proteome maintenance in microalgae under low photon flux [75]. In contrast, under conditions of plenty, deleterious mutations (i.e., having negative *s*) in a population of organisms will be less selected against and thus tend to be fixated more frequently, causing a shift in the stability distribution. In other words, the resource level of the habitat becomes an important parameter in the evolution in the same manner as the temperature.

## Discussion

The derived model has been shown above to provide evolutionary selection pressure of significance enough to shape proteome properties, and the model produces variations in selective pressure that can explain experimentally observed correlations between protein abundance, evolutionary rate, size, and synthetic cost. It reveals new features such as the fundamental nature of previously proposed fitness costs [16], the interplay between and relative importance of protein properties, and the unification of functional and “biophysical” [22] mutations. Although we disregarded epistasis and only considered one protein property to change at the time, the general form of the model (Equation 12) can directly account for epistasis and incomplete compensatory expression by adjusting the parameters of the mutated protein and related proteins in the mutant proteome accordingly. Some implications of the model and their relation to empirical findings are summarized in Table 2. Below, we discuss additional consequences of the model that can explain experimental observations.

**Table 2:** Implications of the model relating to experimental observations.

Model implications	Reason	Observed empirically
Abundant proteins are on average more evolutionary conserved	Equation 13: *s*_i_ ∝ *A*_i_	Ref 19, 20, 21
Bias for lower synthetic cost in proteomes	Equation 13: *s*_i_ ∝ *C_s_*_i_	Ref 30, 31, 32, 33, 76, 77
Bias for lower synthetic cost in particular in abundant and large proteins	Equation 13: *s*_i_ ∝ *C_s_*_i_×*A*_i_×*N*_aai_	Ref 31, 78
Misfolded proteins have a fitness cost	Equation 17: *s*_i_∝Δ*U*_i_	Ref 15, 16, 22
Thermophilic proteins are on average, all else being equal, more stable	Equation 18: T scales down Δ*G*_i_ and increases *U*_i_ and its costs	Ref 68, 71, 72
Abundant proteins are on average, all else being equal, more stable	Equation 18: Proteostatic selection to minimize *U*_i_	Ref 22
Less expression of large proteins	Equation 13: *s*_i_ ∝ A_i_×N_aai_	Ref 49, 66, 79
Trade-offs between stability, proficiency, and cost (e.g. thermophiles have more cystines despite their cost)	Equation 12/13: Couplings between *A*_i_, *k*_di_, *C*_si_, *C*_di_, and Δ*G*_i_	Ref 33, 81
Epistasis	Equation 12: Parameters for protein *j* change upon mutating *i*	Ref 4, 5

First, the factorization of protein properties in our model (Equation 15) implies coupling of these properties during evolution. While it is known that proteomes are biased towards reduced synthetic cost per amino acid [30], [31], [76], [77] viz. the selective advantage of cheaper amino acids, it has also been shown that bias towards cheaper amino acids correlates with both protein size and abundance [31], [78]; this observation is explained by the 2*A*_i_×*N*_aai_×*k*_di_×*C*_si_ product in our model. As *A*_i_ spans five orders of magnitude in yeast [17], [19], abundant proteins will be under much stronger selection, explaining why evolutionary conservation correlates most strongly with expression/abundance levels among several properties. Correspondingly, the significantly lower expression of large proteins [49], [66], [79] is understandable from our model since proteostatic maintenance costs scale with *A*_i_×*N*_aai_. Also the observation that protein stability tends to increase with chain size [50], [80] can be partly rationalized by our model as not due to the physics of protein size (many small proteins are highly stable) but due to selection for stability in larger and more highly expressed proteins.

Although more computational work and more experimental tests are need to fully understand these mechanisms, the property-coupling in our model may explain several anomalies relating to proteome adaptation, such as the observation that cysteine is not selected for cost in most proteomes [33]. This can be explained if the cost reduction due to stability of cysteine bridges out-weights the disadvantage of its higher precursor cost, i.e. a trade-off between *C*_si_ and Δ*G*_i_. Similarly, less selection for precursor cost in thermophiles [77] is understandable from the same type of *C*_si_−Δ*G*_i_ trade-off in favor of more thermostable proteins. Finally, the observed stability-function trade-offs relevant to both natural and laboratory evolution [81] can be partly explained by our model: In future work, we will look at such couplings and how they may have contributed to the shaping of proteomes.

Proteins are marginally stable even if no selection acts on stability itself, due to the mutation-selection balance between the drift towards destabilization caused by the majority of randomly arising mutations and the explicit selection towards maintaining stability at a level that does not undermine fitness [82]. In our model, we have identified a contribution to the selection pressure that constantly works against the random, destabilizing drift: It is, at least partly consistent with minimization of proteostatic costs. Also, intrinsically disordered proteins are not avoiding description by the model as they will also possess both functional, less functional, and nonfunctional states, even if the terms folding and misfolding may be less applicable, giving similar proteostatic consequences. Also, the role of chaperones beyond the initial correct folding of the peptide chain may include a refolding strategy to reduce the cost of compensatory costly degradation and synthesis.

There are several ways to test the validity and range of the model. For example, the resources available in the environment, which limit the metabolic rate, should affect the proteostatic selection pressure since a scarce environment and associated lower metabolic rates would increase selection for low maintenance costs. Such a test requires careful analysis of homologous proteins in variable habitats. Recent analysis of yeast suggests that adaptation towards lower biosynthetic costs indeed occurs during low-resource stress [83]. At the organism level, the experience with cell cycles and dormant states suggest that low resources will cause even a single cell line to switch off reproduction, pointing to the profound link between energy availability and reproduction strategies. Finally, the disposable soma theory is very much a manifestation of multiple observations linking increased energy availability to shorter life times and higher reproductive levels, consistent with our model in which excess chemical energy is ultimately proportional to reproduction and hence, fitness.

## Conclusions

While many selection pressures are likely to act on a protein, shaping the differences seen across protein classes, the overall trends of proteomic properties point to universal components of the selection pressure [6]–[8], [11]–[16]. As a notable example, protein concentrations are under strong selection pressures even in primates [47] and, together with stability, in diving mammals [84]. We have described in this work a selection pressure acting to minimize proteostatic maintenance costs that can explain this observation and a range of other empirical trends in proteomic data. Notably, the corresponding fitness function scales with the remaining proteostatic energy available for reproducing the organism, which is intuitively appealing. Using simple kinetics of protein turnover and thermodynamics of protein folding at steady state, the model recapitulates correlations between evolutionary rates, protein synthesis cost, abundance, size, and stability, and provides simple and universal explanations for the fitness cost of typical mutations, both those affecting function and stability.

The model explains why most typical mutations that slightly impair function or stability are selected against, not necessarily due to compromised cell function but also via the proteostatic cost of compensatory higher protein expression. It shows that selection against protein misfolding (or generally: nonfunctional states of a protein) is consistent with increased proteostatic energy costs of handling such misfolded protein copies, in agreement with recent findings in *E. coli* [44]. The model also provides a framework for understanding and relating biases in precursor ATP cost, protein size, stability, and abundance to organism temperature, habitat resources, and metabolic rates. The interplay between protein properties not previously combined allows modeling of trade-offs, epistasis, co-evolution of properties, and compensatory expression, and provides a mechanism for understanding empirically observed stability-function trade-offs [81]. Once the protein-specific parameters *A*_i_, *N*_aai_, *k*_di_, *C*_si_, *C*_di_, and Δ*G*_i_ are collected for specific proteins, the model may help to understand the evolution and the cellular importance of such individual proteins.
